# Exploratory Evaluation of Refractometric, Biochemical, and Body Weight Change Parameters for Diagnosing Failure of Passive Transfer in Puppies

**DOI:** 10.3390/vetsci13101123

**Published:** 2026-10-21

**Authors:** Halime Kara, Yasin Şenel, Ufuk Kaya, Mustafa Güven, Ali Şenol

**Affiliations:** 1 Department of Veterinary Medicine, Vocational School of Health Services, Ankara Yıldırım Beyazıt University, Ankara 06760, Türkiye; 2 Department of Internal Medicine, Faculty of Veterinary Medicine, Kırıkkale University, Kırıkkale 71450, Türkiye; yasinsenel@kku.edu.tr; 3 Department of Biostatistics, Faculty of Veterinary Medicine, Hatay Mustafa Kemal University, Hatay 31030, Türkiye; u.kaya@mku.edu.tr; 4 Department of Veterinary Medicine, Karşıyaka Vocational School, İzmir Bakırçay University, İzmir 35575, Türkiye; 5 Department of Biochemistry, Faculty of Veterinary Medicine, Kırıkkale University, Kırıkkale 71450, Türkiye; alisenol@kku.edu.tr

**Keywords:** canine neonatology, passive transfer immunity, total protein, globulin, Brix refractometry, body weight change

## Abstract

Failure of passive transfer (FPT) is an important determinant of survival in neonatal puppies. Practical indirect indicators that could serve as alternatives to measuring serum immunoglobulin G (IgG) have not yet been adequately characterized. In this exploratory study, 76 Turkish Kangal puppies aged 48 ± 4 h, born to 12 dams (mean age 40.2 months) were evaluated. Serum IgG measured by ELISA was the reference method, with values below 2.3 g/L classified as FPT. Whole-blood, fresh-serum, and frozen–thawed serum Brix values were evaluated together with serum total protein, albumin, globulin, gamma-glutamyl transferase, canine parvovirus antibody levels, and percentage change in body weight from birth to sampling, using receiver operating characteristic analysis. FPT was identified in six puppies (7.9%). The area under the curve (AUC) values for total protein, globulin, and percentage change in body weight were 0.986, 0.981, and 0.948, respectively, with the associated cutoffs considered exploratory; each detected all six cases with 82.9–94.3% specificity. Fresh-serum Brix showed moderate discrimination (AUC: 0.771), whereas whole-blood Brix was inconclusive (AUC: 0.432). Total protein, globulin, and early body weight change are promising screening indicators for detecting FPT. However, because of the limited number of cases, their cutoffs should be validated in larger populations, including other breeds, before clinical use.

## Simple Summary

Newborn puppies depend largely on antibodies acquired from colostrum for protection against infections. When insufficient amounts of antibodies are absorbed, puppies may become more vulnerable to disease during the first weeks of life. However, direct measurement of antibodies may not always be available in the field. In this study, 76 puppies approximately two days of age were evaluated to identify simple measurements that could indicate inadequate antibody transfer. Six puppies had antibody concentrations below the accepted threshold. The total amount of protein in serum, globulin-a group of proteins that includes antibodies- and percentage change in body weight during the first two days were the indicators that best distinguished puppies with inadequate antibody transfer from those with adequate transfer. Measurements performed using a refractometer, which estimates dissolved substances in blood or serum, were less informative and results obtained from whole blood were inconclusive. Although these findings are preliminary, as inadequate antibody transfer was found in only six puppies, serum total protein, globulin and early weight gain could, if confirmed in larger studies, offer a quick and low-cost way to identify puppies that need extra care or confirmatory antibody testing under field conditions, giving veterinarians and breeders a better chance to intervene early and reduce neonatal losses.

## 1. Introduction

The neonatal period in dogs is a critical stage characterized by physiological adaptation after birth and a markedly higher risk of mortality than during later stages of life [1,2]. Stillbirth and early neonatal mortality have been shown to be multifactorial problems associated with breed, litter size, maternal age, and parturition conditions [1]. Low birth weight has also been associated with mortality, particularly during the first two days of life, whereas insufficient body weight gain during the first 48 h has been associated with losses occurring between the second day of life and weaning [2]. The parturition process, humoral protection, and early growth dynamics should therefore be evaluated together in relation to neonatal survival [2,3].

Because transplacental IgG transfer is limited in dogs, neonatal puppies acquire most of their systemic maternal IgG through colostrum after birth [4,5]. Failure of passive transfer may occur when colostrum is ingested too late or in an insufficient amount, when colostral immunoglobulin content is inadequate, or when intestinal absorption is impaired during the limited period of neonatal permeability. In puppies, a serum IgG concentration below 2.3 g/L at approximately two days of age has been proposed as a clinically relevant definition of FPT [3]. Failure of sufficient transfer of colostrum-derived IgG into the systemic circulation is defined as failure of passive transfer (FPT), for which the clinically relevant threshold in dogs is 2.3 g/L [3]. Adequate IgG transfer has been reported to be important but not sufficient alone for neonatal survival [6], while the pathogen-specific protection provided by colostrum-derived antibodies directly affects the risk of neonatal infection [7,8]. The kinetics of maternally derived canine parvovirus type 2 (CPV-2) antibodies in puppies indicate an association between protective antibody levels and resistance to infection [7], with fewer infections and deaths reported following exposure [8]. Although IgG measurement remains the principal reference method, these findings demonstrate the importance of pathogen-specific serology in the assessment of passive immunity [7,9].

Serum IgG can be measured directly by ELISA; however, the need for laboratory infrastructure, reagents, sample processing, and analysis time may hinder rapid individual decision-making under field conditions where parturition occurs [3,10]. In other animal species, this need has prompted the investigation of indicators that do not directly measure IgG but change following colostrum intake, including serum total protein, globulin, GGT activity, refractometric total solids, and early body weight change [9,10,11]. The available options for evaluating passive transfer and indirectly determining IgG concentrations after colostrum intake in neonatal puppies remain limited. Although a wide range of standardized methods are routinely used in other animal species, promising correlations have been reported in puppies between serum IgG concentrations and the physiological increases in serum gamma-glutamyl transferase (GGT) and alkaline phosphatase (ALP) activities, daily body weight change, and CPV-2-specific antibody titers [2,9,11].

In neonatal dairy calves, serum Brix percentage is known to be a practical test routinely used to assess passive transfer of immunity based on reference serum IgG concentrations [10]. Comparisons of different serum-harvesting methods and refractometer types have demonstrated that the effects of sample-preparation procedures and device characteristics should also be considered during method validation [12]. In practice, serum samples cannot always be analyzed immediately and are often stored frozen for delayed testing, batch analysis, or transport to a central laboratory. Freezing has been reported not to meaningfully affect total protein or Brix readings in calf serum and bovine colostrum [13,14], but this has not been examined in neonatal canine serum. Therefore, the interchangeability of refractometric measurements obtained from fresh whole blood, fresh serum, and frozen–thawed serum requires species- and matrix-specific validation in puppies [10,12]. Our literature search identified no previous study examining these matrices.

The aim of this study was to compare the diagnostic performance of Brix measurements obtained from whole blood, fresh serum, and frozen–thawed serum; serum total protein, albumin, globulin, and GGT concentrations; CPV-2 antibody levels; and percentage body weight change during the first 48 ± 4 h in neonatal puppies, using serum IgG concentration measured by ELISA and the FPT threshold of 2.3 g/L as the reference. Assessing whether a freeze–thaw cycle alters serum Brix measurements is important for determining whether immediate field assessments and delayed laboratory analyses can be used interchangeably. The secondary aim was to evaluate the discriminatory performance of these indicators for identifying FPT using receiver operating characteristic (ROC) analyses and to establish exploratory cutoff values that could be validated in future studies for clinical application.

## 2. Materials and Methods

The study was conducted between April and August 2026 using samples obtained from privately owned dogs and licensed breeding establishments in Ankara and the surrounding region.

### 2.1. Animal Population

The study population consisted of 48 ± 4 h old Turkish Kangal puppies born naturally and without complications to dams with no history or clinical evidence of disease. To be eligible for inclusion, puppies were required to have been born alive, exhibit a suckling reflex, and allow the planned blood sample to be collected safely. Puppies delivered following dystocia or by cesarean section, as well as those that yielded an insufficient sample volume or had missing essential analytical data, were excluded from the study. Puppies that died before the 48 ± 4 h sampling point could not be included. After these exclusions, 76 puppies from 12 dams (mean age, 40.2 months; range, 15–72 months) were included; 3 of the dams were primiparous and 9 were multiparous. All dams had been routinely vaccinated with an annual multivalent canine vaccine that includes protection against CPV-2, and none had a known history of natural CPV-2 infection; their serum CPV-2 antibody titers were not measured.

Each puppy was weighed immediately after birth, before its first colostrum intake, using a calibrated electronic scale (Mastek, SH-125, Suzhou, China). The second weighing was performed concurrently with blood sampling at 48 ± 4 h of age. Both measurements were recorded in grams. Absolute body weight change was calculated by subtracting the initial weight from the second weight, whereas relative body weight change was calculated as a percentage of the initial body weight.

### 2.2. Sample Collection

At 48 ± 4 h of age, a total of 1.0 mL of blood was collected from the jugular vein of each puppy. Immediately after collection and before clot formation, approximately 0.1 mL (about two drops) of this sample was used for the whole-blood Brix measurement, and no additional blood was collected for this purpose. The remaining blood was transferred into serum separator tubes containing no anticoagulant. Following clot formation, the blood samples were centrifuged at 3000 rpm for 10 min using a portable microcentrifuge (Nüve Tech., NF 024, Ankara, Türkiye) under field conditions. The separated serum was transferred into sterile microtubes. Samples were transported to the laboratory under a maintained cold chain and stored at −20 °C until analysis.

### 2.3. Refractometric Measurements

Refractometric measurements were performed using the same digital Brix refractometer (Milwaukee Instruments, MA871, Rocky Mount, NC, USA). Separate measurements were obtained from uncentrifuged fresh whole blood, fresh serum, and frozen–thawed serum for each puppy, and the results were recorded as Brix percentages (%). Whole-blood refractometric measurement was performed by applying venous blood directly to the measurement surface of the device immediately after collection, before clot formation. Fresh-serum Brix measurements were performed after serum separation at the end of centrifugation, whereas frozen–thawed serum samples were measured before laboratory analyses. For each analysis, approximately 0.1 mL (about two drops) of sample was applied to the prism/measurement surface of the device, and the value was recorded after stabilization. Measurements were performed in accordance with the manufacturer’s instructions. After each sample, the measurement surface was cleaned with alcohol and dried, and calibration was checked using distilled water.

### 2.4. Serum IgG and CPV Antibody Measurements

Serum total IgG concentrations were determined using a commercial ELISA kit (Canine IgG ELISA, ab157701, Abcam, Cambridge, UK) in accordance with the manufacturer’s instructions. Before analysis, all kit reagents were brought to room temperature, and serum samples were diluted 1:100,000 using the kit diluent in two sequential steps, as recommended by the manufacturer for canine serum. Standards and diluted samples (100 µL each) were added to the microplate wells, and the plate was covered and incubated for 15 min at room temperature. After four washes, 100 µL of 1× enzyme–antibody conjugate was added to each well, followed by a second 15 min incubation at room temperature in the dark. The wells were washed four more times, and 100 µL of TMB substrate was then added and left for 10 min at room temperature in the dark. The reaction was stopped by adding 100 µL of stop solution to each well. Quantitative IgG concentrations in the samples were calculated from a calibration curve generated using standards of known concentrations with a four-parameter logistic algorithm (4PL), and the final values were obtained by multiplying the results by the applied dilution factor. Optical density (OD) values were measured spectrophotometrically at a wavelength of 450 nm using a Multiskan GO microplate reader (Thermo Fisher Scientific, Waltham, MA, USA).

Serum CPV-2 antibody levels were similarly determined using an ELISA kit (PARVO IgG Ab ELISA, Agrolabo SpA, Scarmagno, Italy) in accordance with the manufacturer’s instructions. All kit reagents were brought to room temperature (20–25 °C) before use, and serum samples were diluted 1:100 in the kit diluent. Positive and negative controls and diluted samples (100 µL each) were added to the antigen-coated wells, and the plate was covered and incubated for 10 min at room temperature. After three washes, 100 µL of conjugate was added to each well, and the plate was incubated for a further 10 min. Following five washes, 100 µL of TMB substrate was added and left for 5 min at room temperature, after which the reaction was stopped with 100 µL of stop solution per well. Based on the data obtained, samples with S/*P* values (sample OD/positive control OD) > 0.15 were considered to have protective antibody titers, and specific antibody titers were calculated from the sample-to-positive-control ratio (S/*P*) using the formula specified by the manufacturer (CPV-2 IgG Ab titer = 54[e4x]). Optical density (OD) values were measured spectrophotometrically at a wavelength of 450 nm using a Multiskan GO microplate reader (Thermo Fisher Scientific, Waltham, MA, USA).

### 2.5. Biochemical Analyses

Serum total protein (TP, catalog no. C4500650A), albumin (ALB, catalog no. C1200620A), and gamma-glutamyl transferase (GGT, catalog no. C3500620A) were measured. Measurements were performed using an automated biochemistry analyzer (Gesan, Chem200, Campobello di Mazara, Italy) with the manufacturer’s reagents and standard operating procedures. Serum globulin concentrations were calculated as the difference between total protein and albumin concentrations.

### 2.6. Reference Definition and Study Variables

Serum IgG concentration measured by ELISA was accepted as the reference measure of passive transfer status. Puppies with serum IgG concentrations < 2.3 g/L were classified as having FPT, whereas those with concentrations ≥ 2.3 g/L were classified as having adequate passive transfer [3]. The primary index tests were Brix measurements obtained from whole blood, fresh serum, and frozen–thawed serum. Absolute and percentage body weight changes, serum total protein, albumin, globulin, and GGT values, and continuous and categorical CPV antibody results were evaluated as secondary indirect markers.

### 2.7. Statistical Analysis

The pre-study sample size was calculated based on a comparison of continuous parameters between two independent groups of puppies with and without FPT. Assuming a two-sided type I error probability of 0.05, a target power of 0.80, and a standardized effect size of d = 0.82, a minimum of 76 puppies was determined to be required. Calculations were performed using PASS 11 and G*Power 3.1.9.2 software [15,16,17]. This calculation was not designed to ensure adequate precision for ROC-based estimates or cutoff determination, and the study was therefore not powered as a confirmatory diagnostic accuracy study. A post hoc sensitivity analysis for the observed group sizes (6 vs. 70) showed that the minimum detectable effect size for the Mann–Whitney U test was d = 1.28 (two-sided α = 0.05, power = 0.80); post hoc power exceeded 85% for total protein, globulin, and body weight change but was approximately 45–55% for GGT and fresh-serum Brix.

Each puppy was considered an independent unit of analysis. Clustering according to dam or litter was not modeled. Before analysis, the dataset was checked for range, consistency, and missing values. Because of the limited sample size of the FPT group and the distributional characteristics of the data, continuous variables were summarized as the median and interquartile range, whereas categorical variables were summarized as numbers and percentages.

Continuous variables were compared between the FPT and adequate passive transfer groups using the Mann–Whitney U test. Sex and categorical CPV antibody status were evaluated using Fisher’s exact test.

Associations between serum IgG concentrations and indirect markers were examined using Spearman’s rank correlation coefficient (ρ), which was preferred over Pearson’s correlation because several variables were not normally distributed, IgG concentrations spanned a wide range with extreme values, and the relationships were expected to be monotonic rather than strictly linear. These correlations were considered exploratory and were not interpreted as evidence of causal relationships. Agreement among paired Brix measurements from whole blood, fresh serum, and frozen–thawed serum was evaluated using Bland–Altman analysis. For each pair, the mean difference (bias), its 95% confidence interval, and the 95% limits of agreement (mean difference ± 1.96 × standard deviation of the differences) were calculated. Bland–Altman plots were used to examine systematic bias and whether differences varied with measurement magnitude, informing the assessment of whether Brix results from the three sample matrices could be used interchangeably. Proportional bias was formally assessed by linear regression of the paired differences on the paired means. Because no clinically acceptable limits of agreement have been established for Brix refractometry in neonatal puppies, the clinical relevance of the limits of agreement was judged by comparing their width with the proximity of measurements to the data-derived fresh-serum Brix cutoff.

The ability of each index test to discriminate FPT was evaluated using ROC analysis. FPT was defined as the positive class, and the area under the curve (AUC) and its 95% confidence interval were calculated. The optimal cutoff was defined as the point that maximized the Youden index (sensitivity + specificity − 1). Sensitivity and specificity were calculated at this cutoff, together with their exact (Clopper–Pearson) 95% confidence intervals. Because of the small size of the FPT group, the cutoffs and performance measures were considered exploratory.

All tests were two-sided, *p* < 0.05 was considered statistically significant. Given the exploratory design of the study, no adjustment for multiple comparisons was applied, and *p* values were interpreted descriptively. Analyses were performed in Python 3.12.13 using NumPy 2.3.5, pandas 2.2.3, SciPy 1.17.0, and scikit-learn 1.8.0.

## 3. Results

After excluding dogs with missing study data or those meeting the exclusion criteria, 76 puppies were included in the study. Dam and puppy identifiers were not included in the analyses. Using serum ELISA IgG < 2.3 g/L as the reference definition, 6 of the 76 puppies were classified as having failure of passive transfer (FPT; 7.9%; exact 95% CI, 3.0–16.4), whereas adequate passive transfer was identified in 70 of the 76 puppies (92.1%). The six FPT cases came from three litters: three puppies from one litter, two from another, and one from a third. The median serum IgG concentration was 1.62 g/L (IQR, 1.12–1.94) in the FPT group and 18.18 g/L (IQR, 11.37–23.91) in the adequate passive transfer group (Table 1).

The study included 35 female and 41 male puppies. FPT was identified in 4 of 35 females (11.4%) and 2 of 41 males (4.9%). There was no evidence of a sex-related difference (Fisher’s exact *P* = 0.405; Table 2). CPV-2 antibody levels were measured in all 76 puppies; 60 (78.9%) had protective levels (S/*P* > 0.15), whereas the remaining 16 did not exceed the protective threshold. The proportion of puppies exceeding the protective antibody titer threshold was lower among puppies with FPT (2/6, 33.3%) than among those with adequate passive transfer (58/70, 82.9%; Fisher’s exact *P* = 0.016; Table 2).

**Figure 1:**
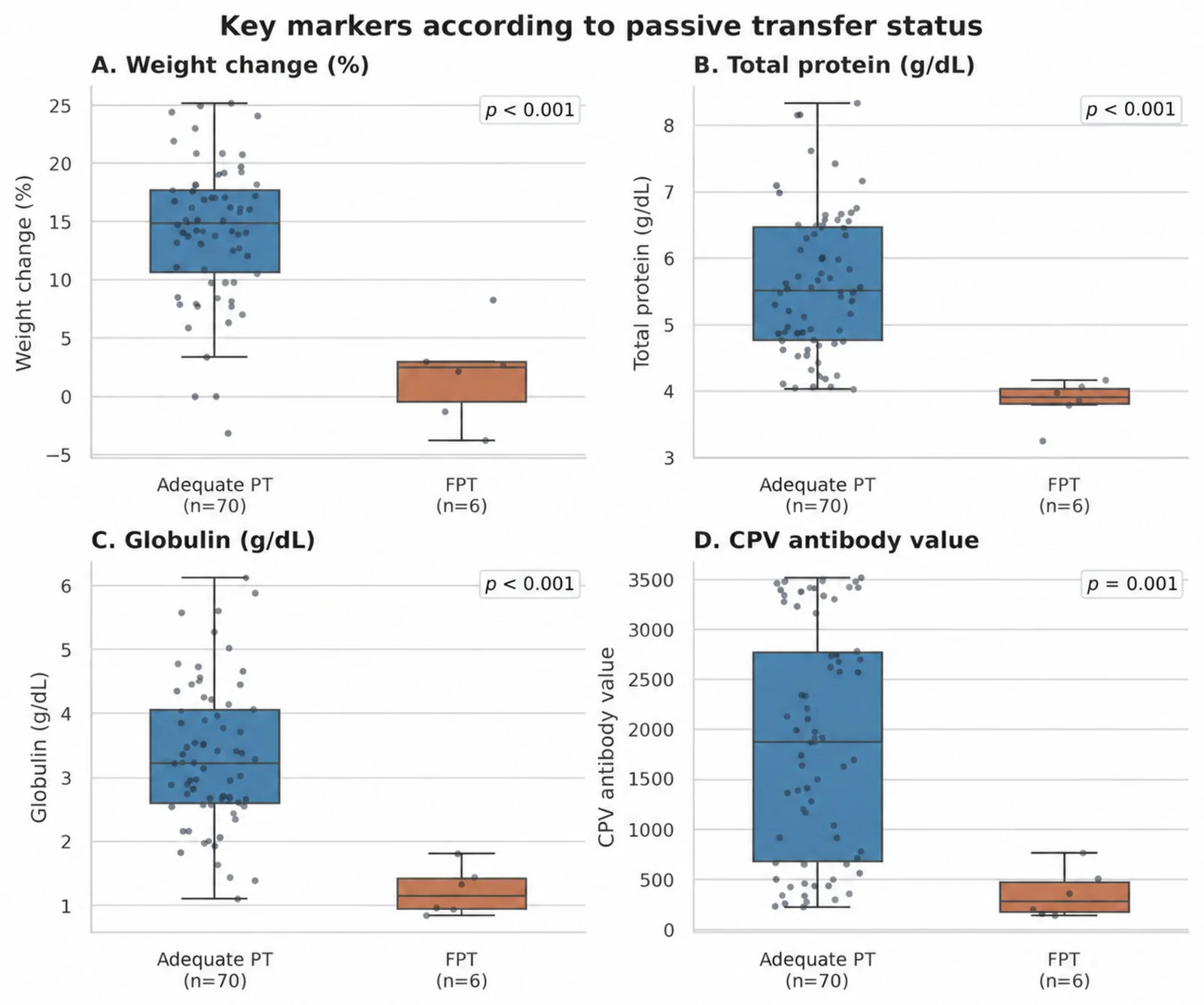
Key indirect markers according to passive transfer status. Boxes show the interquartile range and median; individual puppies are displayed as points. FPT, failure of passive transfer; PT, passive transfer.

Early body weight change differed markedly according to passive transfer status (*p* < 0.001). Puppies with adequate passive transfer had a median body weight gain of 81.0 g (IQR, 62.3–100.0), corresponding to 14.84% (IQR, 10.61–17.64) of their initially recorded body weight. In contrast, puppies with FPT had a body weight gain of 14.0 g (IQR, −3.0 to 16.0), corresponding to 2.43% (IQR, −0.48 to 2.92). The distributions of percentage body weight change are presented in Figure 1A.

**Figure 2:**
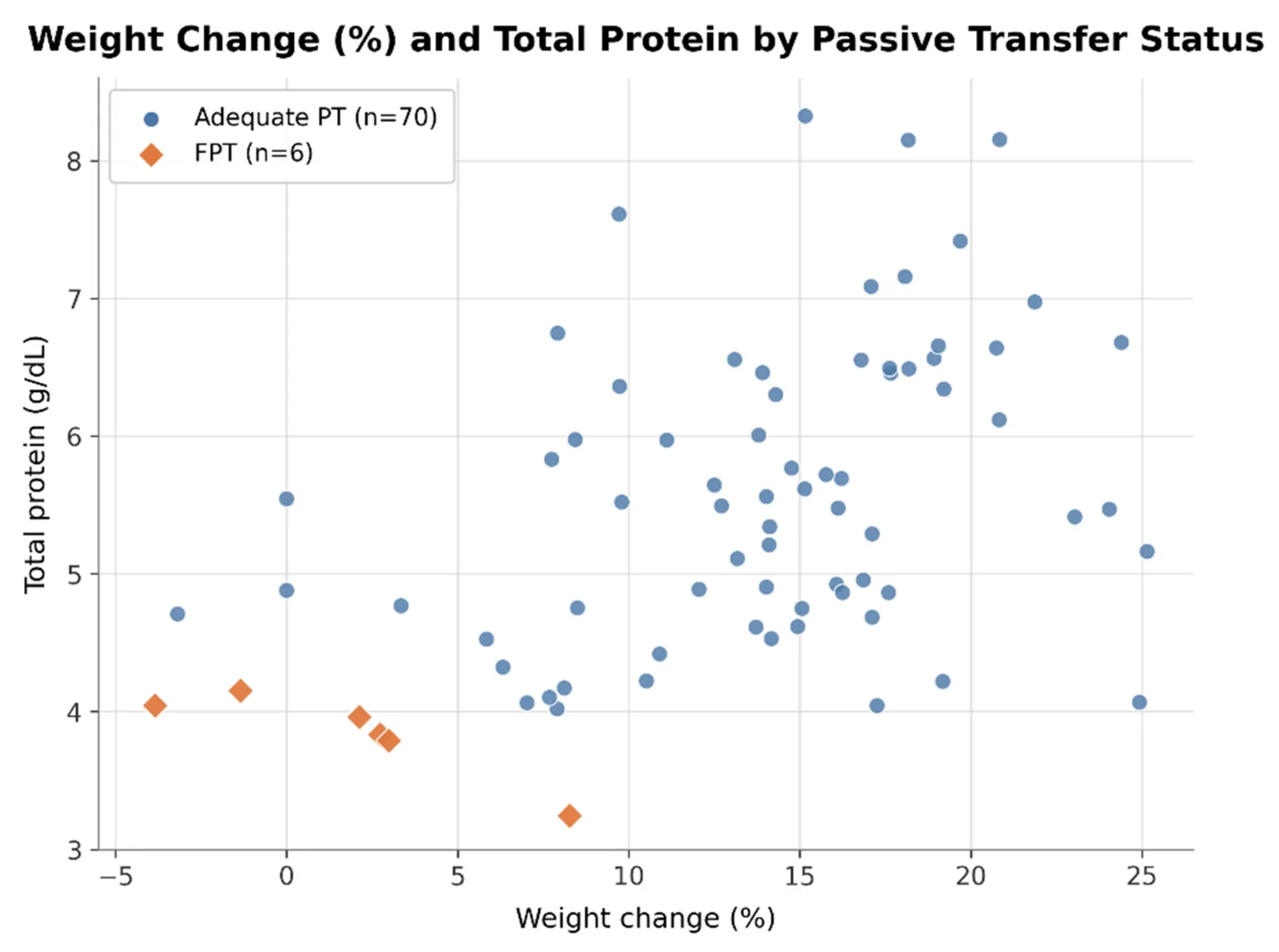
Relationship between percentage body weight change and serum total protein according to passive transfer status. Each symbol represents one puppy. Puppies with serum immunoglobulin G concentrations < 2.3 g/L were classified as having failure of passive transfer. FPT, failure of passive transfer; PT, passive transfer.

Total protein and globulin showed the largest differences between the groups. Median total protein was 5.51 g/dL in the adequate passive transfer group and 3.90 g/dL in the FPT group (*p* < 0.001), whereas median globulin was 3.22 and 1.14 g/dL, respectively (*p* < 0.001). CPV antibody assay values were also lower in puppies with FPT (median, 271.9 vs. 1869.4; *p* = 0.001). Fresh-serum Brix and albumin values differed between the groups (*p* = 0.029 and *p* = 0.026, respectively). No differences were observed between the groups in whole-blood Brix, frozen–thawed serum Brix, or GGT values (Table 1; Figure 1B–D). The joint distribution of percentage body weight change and serum total protein concentration according to passive transfer status is shown in Figure 2.

**Figure 3:**
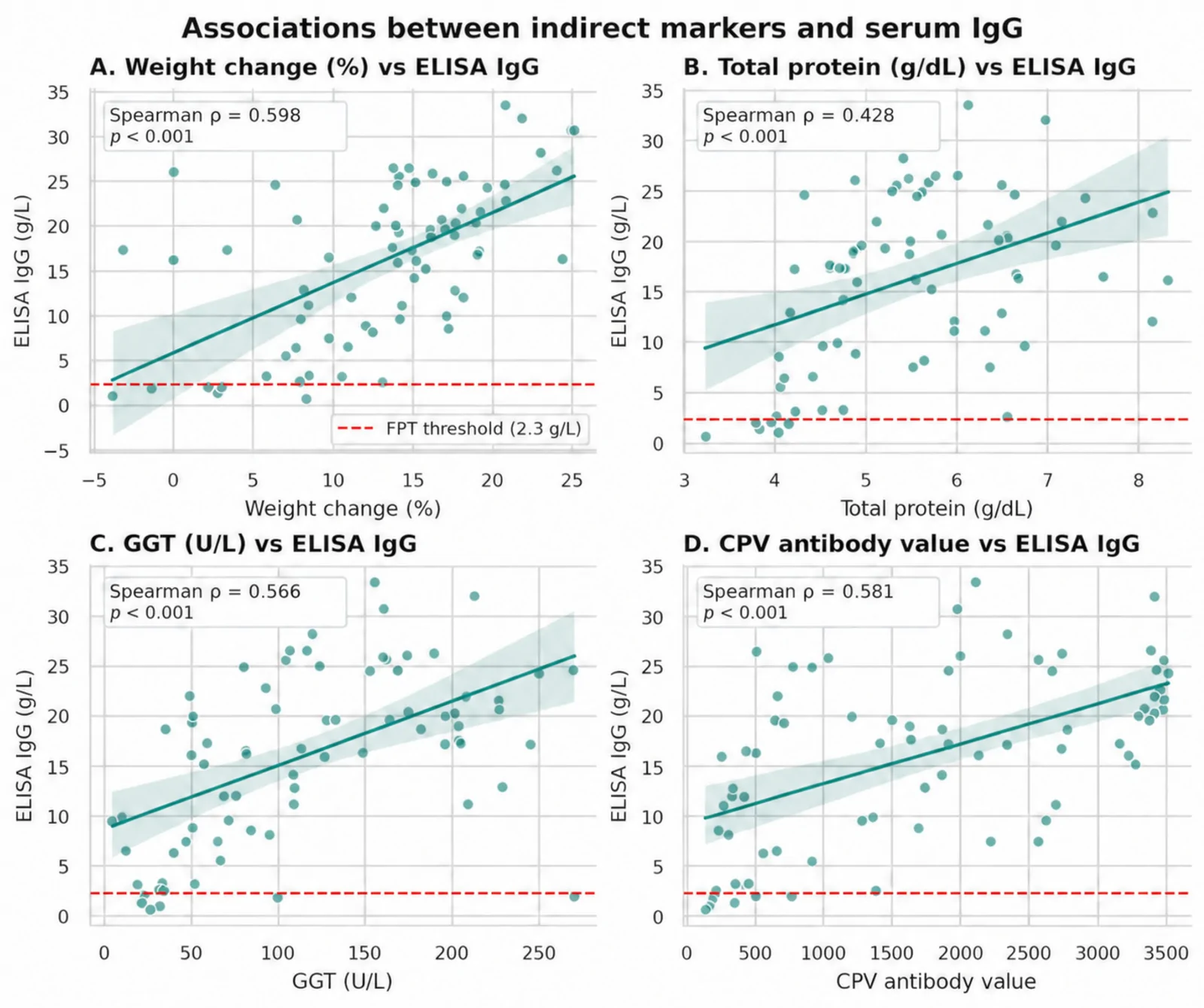
Associations between selected indirect markers and serum ELISA IgG. Solid lines represent linear fitted trends with 95% confidence bands and are shown for visual guidance only; the dashed red line indicates the FPT threshold of 2.3 g/L. Associations were quantified using Spearman’s rank correlation; coefficients are shown with *p* values. The shaded area represents the 95% confidence interval, and the dots represent individual data points.

Serum IgG showed a moderate positive correlation with percentage body weight change (Spearman’s ρ = 0.598, *p* < 0.001). This was followed, in descending order, by the CPV antibody assay value (ρ = 0.581, *p* < 0.001), GGT (ρ = 0.566, *p* < 0.001), absolute body weight change (ρ = 0.515, *p* < 0.001), globulin (ρ = 0.473, *p* < 0.001), and total protein (ρ = 0.428, *p* < 0.001). Albumin was inversely correlated with IgG (ρ = −0.367, *p* = 0.001) (Table 3). Selected relationships and the FPT threshold of 2.3 g/L are shown in Figure 3.

**Figure 4:**
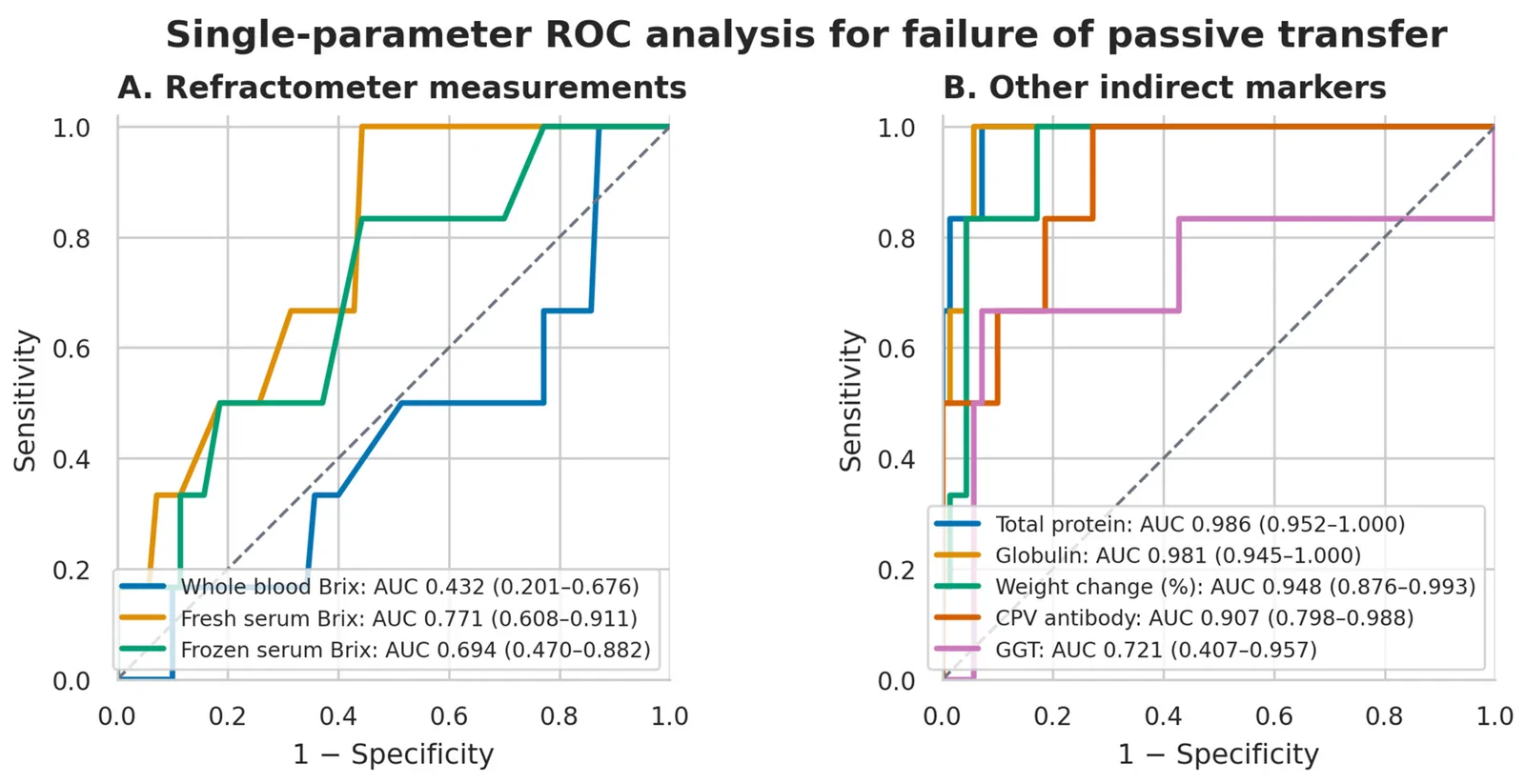
Receiver operating characteristic curves for individual predictors of failure of passive transfer. Lower marker values were defined as indicating FPT. The dashed diagonal line represents the reference line of no discrimination.

Among the individual markers, total protein provided the best discrimination of FPT (AUC, 0.986; 95% confidence interval, 0.952–1.000), followed closely by globulin (AUC, 0.981; 95% CI, 0.945–1.000) and percentage body weight change (AUC, 0.948; 95% CI, 0.876–0.993). The CPV antibody assay value also demonstrated high discriminatory performance (AUC, 0.907; 95% CI, 0.798–0.988). The exploratory cutoff values determined using the Youden method were ≤4.15 g/dL for total protein, ≤1.80 g/dL for globulin, ≤8.27% for body weight change, and ≤761.0 for the CPV antibody assay value. All four cutoffs provided 100% sensitivity, with specificities of 92.9%, 94.3%, 82.9%, and 72.9%, respectively (Table 4; Figure 4). Because these sensitivity estimates were based on the detection of all six FPT cases, they corresponded to an exact 95% confidence interval of 54.1–100%, indicating substantial uncertainty.

Fresh-serum Brix had an AUC of 0.771 (95% CI, 0.608–0.911) and performed numerically better than frozen–thawed serum Brix (AUC, 0.694; 95% CI, 0.470–0.882) and whole-blood Brix (AUC, 0.432; 95% CI, 0.201–0.676). At the data-derived cutoff of ≤7.80%, fresh-serum Brix provided 100% sensitivity but only 55.7% specificity. GGT demonstrated an AUC of 0.721 (95% CI, 0.407–0.957); a cutoff of ≤31.5 U/L provided 66.7% sensitivity and 92.9% specificity (Table 4; Figure 4).

**Figure 5:**
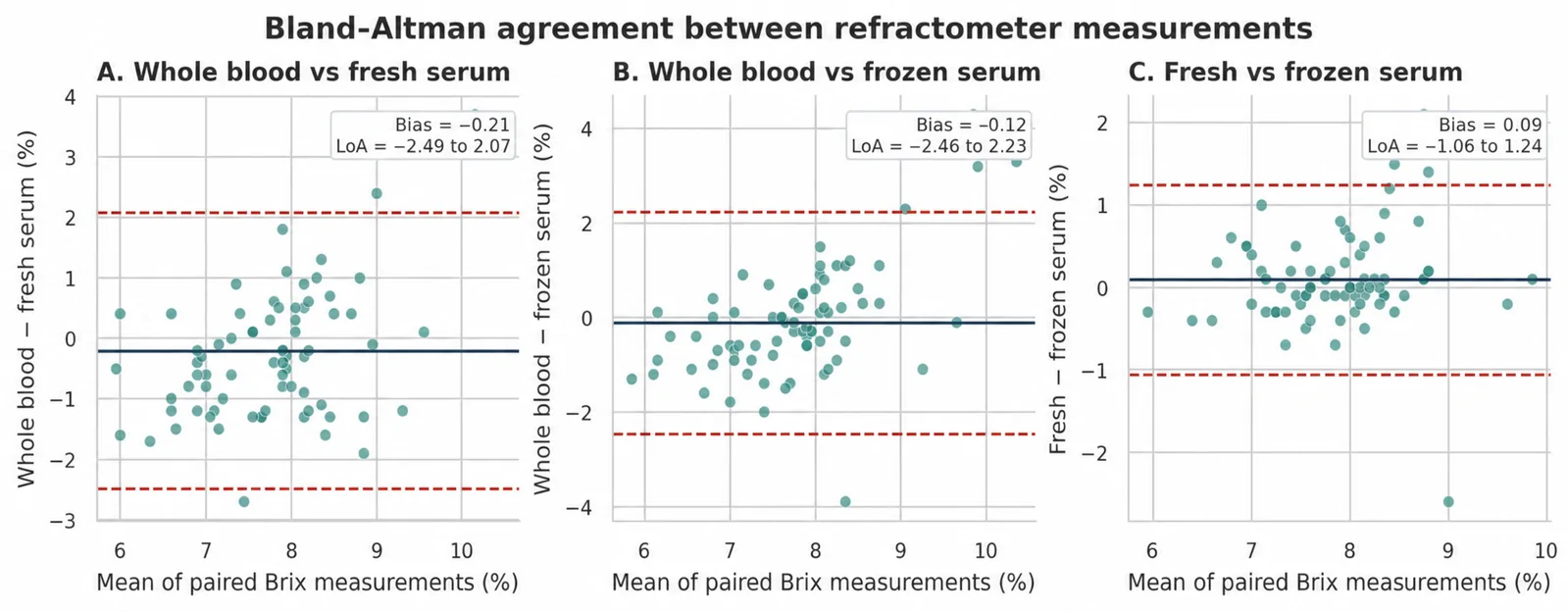
Bland–Altman plots comparing paired Brix measurements. Solid lines indicate mean bias and dashed lines indicate the 95% limits of agreement. The dots represent individual paired data points.

The mean differences among the three refractometric measurements were small, although the limits of agreement varied according to the methods compared (Table 5; Figure 5). Whole-blood Brix was, on average, 0.21 percentage points lower than fresh-serum Brix (95% limits of agreement, −2.49 to 2.07) and 0.12 percentage points lower than frozen–thawed serum Brix (−2.46 to 2.23). Fresh-serum Brix was, on average, 0.09 percentage points higher than frozen–thawed serum Brix and exhibited the narrowest limits of agreement (−1.06 to 1.24) with no evidence of proportional bias (regression slope of differences on means, *p* = 0.384). Therefore, despite the small mean biases, the serum measurements showed closer pairwise agreement with each other than either did with whole-blood Brix.

## 4. Discussion

In this study, total protein, globulin, and percentage body weight change emerged as the most prominent indicators for discriminating failure of passive transfer (FPT), as defined on the basis of serum IgG concentrations measured by ELISA. This is biologically plausible, as globulin and total protein directly reflect absorbed immunoglobulins [18] and early weight gain reflects colostrum intake [9], whereas the other markers are influenced by additional factors discussed below. In contrast, fresh-serum Brix showed more limited discriminatory performance, whereas the performance of whole-blood Brix remained inconclusive. However, these results were based on only six FPT cases. Therefore, the findings should be interpreted as the identification of candidate indicators that can be tested in independent studies rather than the validation of a diagnostic algorithm ready for clinical use [19]. If validated, these markers would not replace IgG measurement but would help identify puppies needing confirmatory testing or closer monitoring when direct testing is unavailable. Since a missed FPT case carries greater risk than an unnecessary test, screening thresholds should favor sensitivity. With only six FPT cases and no outcome data, however, the present thresholds are not yet suitable for guiding clinical decisions.

The detection of FPT in 6 of the 76 puppies using the criterion of serum IgG < 2.3 g/L corresponded to a prevalence of 7.9% in this sample (95% confidence interval, 3.0–16.4%; Table 1). The threshold used (2.3 g/L) was consistent with other studies in the literature; however, the proportion of inadequate passive transfer identified in the present study was lower than that reported in other studies [3,9]. The numerically lower prevalence observed here does not, by itself, demonstrate superior breeding conditions or a true biological difference between populations, because inclusion criteria and environmental conditions were not evaluated using a common comparative design.

In a field study, IgG concentrations were above 2.3 g/L in all 104 Kangal puppies from which blood samples could be collected, although preweaning losses continued to occur. Moreover, 34 of the 138 puppies born were lost before sampling [6]. Considered together with the present study, these findings demonstrate that the prevalence of FPT measured among puppies available for sampling and the neonatal health status of the entire birth cohort are not equivalent concepts. Particularly in assessments that do not include losses occurring before sampling, a low prevalence of FPT should not be interpreted as indicating that all puppies are at low risk [6].

The markedly lower total protein and globulin concentrations in the FPT group were among the important findings in this sample. The AUC was 0.986 for total protein and 0.981 for globulin. This finding is thought to reflect the direct contribution of immunoglobulins absorbed from the intestine, depending on colostrum quality and intake volume, to the serum protein profile. Our findings support the physiological validity of total protein and globulin for detecting hypoglobulinemia, which is a direct consequence of FPT [3,18]. Although these indirect methods have frequently been investigated in other species, no other study evaluating total protein and globulin concentrations for determining passive transfer success in dogs was identified [18,20]. In this respect, these variables are considered important parameters that should be investigated in future studies.

The refractometric findings indicate that the high diagnostic accuracy reported in the calf literature should not be extrapolated directly to puppies. In a study of 400 calves aged 3–6 days, a strong correlation was reported between serum Brix values and IgG concentrations measured by radial immunodiffusion, with 88.9% sensitivity and 88.9% specificity obtained at a Brix threshold of 8.4% [10]. Several other studies in the literature have also demonstrated this strong correlation [21,22,23]. In the present study, however, the correlation between fresh-serum Brix and IgG was 0.180, the AUC was 0.771, and specificity at the selected cutoff of ≤7.80% was 55.7%. Because of differences in species, age range, reference IgG method, and the definition of FPT, the thresholds from these studies were not considered directly comparable. In particular, applying the Brix decision threshold developed for IgG < 10 g/L in calves to the classification of IgG < 2.3 g/L in dogs complicates the assessment [10].

Although fresh-serum Brix values were lower in the FPT group (*p* = 0.029), the difference between the groups was small and should be interpreted with caution given the number of comparisons performed. For whole-blood Brix, the AUC was 0.432, with a 95% confidence interval of 0.201–0.676. These results do not support the use of whole-blood measurements under the conditions applied in this study. A calf study that examined paired serum and plasma samples demonstrated that refractometric results may vary according to the sample matrix [24].

The small mean difference between Brix values obtained from fresh and frozen–thawed serum (+0.09 Brix percentage points) initially suggests that the two sample types produce similar results. However, the 95% limits of agreement ranging from −1.06 to +1.24 Brix percentage points in the Bland–Altman analysis indicate that differences capable of affecting clinical classification may occur between measurements in individual puppies. Although no clinically acceptable limits of agreement have been established for Brix refractometry in puppies, these limits are wider than the difference between the median fresh-serum Brix values of the FPT and adequate passive transfer groups (7.35% vs. 7.95%); a disagreement of this magnitude could therefore move a result across the exploratory cutoff of ≤7.80%. Villarroel et al. [13] identified a very strong correlation (r = 0.896) between fresh- and frozen-serum total protein measurements used to evaluate failure of passive transfer in calves and reported that freezing did not produce variation of a magnitude that would interfere with clinical diagnosis. Similarly, Morrill et al. [14] reported that Brix refractometer readings were not statistically affected by multiple freeze–thaw cycles applied to samples and that frozen samples could be used reliably in field and laboratory studies. Elsohaby et al. [24] reported that refractometric measurements may be affected by the sample matrix and that different decision thresholds may be required for serum and plasma. Therefore, the sample matrix and storage conditions should be considered when interpreting Brix measurements, particularly for results close to the decision threshold.

Percentage body weight change provided the most notable discriminatory performance apart from the biochemical indicators. The median change was 2.43% in the FPT group and 14.84% in the adequate passive transfer group; the correlation with IgG was 0.598, and the AUC was 0.948. The use of percentage change instead of absolute gain in grams proportionally standardized the differences in initial body weight among puppies. It was therefore used to reduce the effect of variation in initial body weight. The positive association between body weight change during the first two days of life and IgG was consistent with the literature [3,9]. However, early weight gain may also be influenced by factors unrelated to passive transfer, such as litter size, maternal milk production, competition for teats, and individual nursing behavior, which were not recorded in the present study [2,9]. Although breed was uniform and birth weight did not differ between groups, the association between IgG transfer and early growth should therefore be interpreted with caution rather than as evidence of a direct causal relationship, and percentage body weight change should be regarded as a nonspecific exploratory indicator.

The cutoff of ≤8.27% body weight change identified for FPT in the present study differed markedly from the −2.7% threshold reported by Mila et al. [9]. The higher birth weights of the puppies included in the study and the limited proportion of puppies with FPT were considered potential contributors to this difference. Furthermore, 8.27% is not a universal threshold for normal growth but an exploratory cutoff selected to distinguish the low-IgG group within the present sample.

The positive correlation between serum GGT activity and IgG concentration is biologically consistent with the transfer of colostrum-derived constituents. Neonatal puppies have very low serum GGT activity before suckling, whereas canine colostrum contains high GGT activity originating from the mammary gland. An experimental study demonstrated that serum GGT activity increased during the first 24 h in puppies that suckled colostrum, whereas the same increase did not occur in puppies deprived of colostrum [11]. Nevertheless, GGT showed only moderate discrimination in the present study (AUC, 0.721; 95% CI, 0.407–0.957). Variation in the timing and amount of colostrum intake, together with possible changes in serum GGT activity between the early post-suckling period and sampling at 48 ± 4 h, may have contributed to this finding.

The positive correlation between the quantitative CPV antibody assay result and total IgG (ρ = 0.581) supports an association between overall and pathogen-specific passive transfer. A study reporting a correlation of ρ = 0.71 between total IgG and maternally derived CPV-2 antibody titers on the second day of life also provided evidence in this direction, although it demonstrated that overall FPT and inadequate CPV-2 antibody levels did not completely overlap [9]. Because puppies were sampled at 48 h of age, the dam was the only possible source of CPV-2 antibodies, and each of the 12 litters included at least one puppy with protective antibody levels; differences between littermates are therefore likely to reflect differences in colostral antibody intake. However, serum CPV-2 antibody titers of the dams were not measured, and the level of maternally derived CPV-2 antibodies in a puppy depends on the dam’s own titer as well as on colostral transfer. A low value therefore does not necessarily indicate inadequate total IgG transfer, as illustrated by the 12 of 70 puppies (17.1%) with adequate passive transfer whose antibody levels remained below the protective threshold. The discriminatory performance of this marker may thus not be generalizable to litters from dams with different vaccination or exposure histories.

The findings of this study should be interpreted within the context of several limitations. The inclusion of only six puppies in the FPT group limits the precision of sensitivity, specificity, and the cutoff values obtained from ROC analyses. The post hoc analyses indicated adequate power for the large differences observed in total protein, globulin, and body weight change, but limited power for moderate differences; the lack of significant differences for markers such as Brix and GGT should therefore not be interpreted as evidence of no association with passive transfer. Furthermore, because the ROC cutoffs were both derived and evaluated using the same dataset, the diagnostic performance estimates may be subject to optimism bias. Resampling-based confidence intervals quantify sampling variability but do not constitute external validation, particularly with only six FPT cases. Therefore, the identified thresholds should not be regarded as validated clinical cutoffs but as exploratory thresholds requiring validation in independent and larger populations. Moreover, the 100% sensitivity observed at several cutoffs (exact 95% CI, 54.1–100%) indicates only that all six cases in this sample were detected; it does not exclude missed cases in other populations, and a high AUC alone does not establish clinical benefit. Future studies should therefore define the consequences of false-negative and false-positive classifications and evaluate the net benefit of the proposed screening approach. The failure to statistically model potential clustering among puppies from the same litter may also have affected the precision of the estimates. Because only puppies that survived until the sampling time could be included, puppies that were clinically ill or had not ingested sufficient colostrum, which are the most likely to die within the first 48 h, were probably not represented. This may have resulted in underrepresentation of severe FPT cases and introduced selection or spectrum bias. Because milder cases are generally more difficult to detect, the reported sensitivity estimates are more likely to be conservative than inflated, although the magnitude of this effect cannot be quantified with only six cases.

## 5. Conclusions

In this exploratory study, serum total protein, calculated globulin, and early percentage body weight change showed the highest discriminatory performance for detecting failure of passive transfer (FPT), defined as a serum IgG concentration below 2.3 g/L in puppies. However, because these estimates were based on six FPT cases, they are imprecise and should be regarded as hypothesis-generating rather than confirmatory. Although GGT activity and CPV antibody levels were positively associated with serum IgG, their individual diagnostic performance was more limited than that of total protein, globulin, and percentage body weight change. Serum Brix showed limited discriminatory performance, whole-blood Brix was inconclusive, and fresh and frozen–thawed serum Brix values may not be directly interchangeable. These findings identify candidate indicators for FPT screening in puppies, whose cutoffs should be validated in larger, independent populations before clinical use.

## Figures and Tables

**Table 1:** Continuous variables according to passive transfer status.

Variable	Adequate PT (*n* = 70)	FPT (*n* = 6)	*p* Value
ELISA IgG (g/L)	18.18 (11.37–23.91)	1.62 (1.12–1.94)	—
Body weight, first weighing (g)	579.0 (487.0–639.2)	567.0 (543.5–580.0)	0.644
Body weight, second weighing (g)	660.0 (553.8–741.5)	582.0 (558.5–596.5)	0.104
Weight change (g)	81.0 (62.2–100.0)	14.0 (−3.0–16.0)	<0.001
Weight change (%)	14.84 (10.61–17.64)	2.43 (−0.48–2.92)	<0.001
Whole-blood Brix (%)	7.60 (6.80–8.17)	7.95 (7.22–8.68)	0.589
Fresh-serum Brix (%)	7.95 (7.43–8.30)	7.35 (7.05–7.72)	0.029
Frozen-serum Brix (%)	7.80 (7.50–8.20)	7.50 (7.08–7.70)	0.118
Total protein (g/dL)	5.51 (4.76–6.46)	3.90 (3.80–4.03)	<0.001
Albumin (g/dL)	2.24 (2.09–2.50)	2.51 (2.43–2.77)	0.026
Globulin (g/dL)	3.22 (2.60–4.06)	1.14 (0.94–1.41)	<0.001
GGT (U/L)	111.11 (60.39–174.39)	29.05 (23.52–82.43)	0.075
CPV antibody assay value	1869.4 (674.4–2767.3)	271.9 (169.3–464.8)	0.001

Data are median (interquartile range). Continuous groups were compared using Mann–Whitney U tests.

**Table 2:** Sex and categorical CPV antibody status according to passive transfer status.

Characteristic	Adequate PT, *n*/*N* (%)	FPT, *n*/*N* (%)	*p* Value
Female	31/70 (44.3)	4/6 (66.7)	0.405
Male	39/70 (55.7)	2/6 (33.3)	
CPV antibody positive	58/70 (82.9)	2/6 (33.3)	0.016
CPV antibody negative	12/70 (17.1)	4/6 (66.7)	

*p* values are from Fisher’s exact tests. CPV, canine parvovirus; FPT, failure of passive transfer; PT, passive transfer.

**Table 3:** Spearman correlations between indirect markers and serum ELISA IgG.

Indirect Marker	Spearman ρ	*p* Value
Weight change (g)	0.515	<0.001
Weight change (%)	0.598	<0.001
Whole-blood Brix (%)	−0.013	0.910
Fresh-serum Brix (%)	0.180	0.119
Frozen-serum Brix (%)	0.157	0.176
Total protein (g/dL)	0.428	<0.001
Albumin (g/dL)	−0.367	0.001
Globulin (g/dL)	0.473	<0.001
GGT (U/L)	0.566	<0.001
CPV antibody assay value	0.581	<0.001

CPV, canine parvovirus; GGT, γ-glutamyl transferase.

**Table 4:** Diagnostic performance of individual markers for failure of passive transfer.

Predictor	AUC (95% Bootstrap CI)	*p* Value	Youden Cutoff	Sensitivity (%)	Sensitivity (95% CI)	Specificity (%)	Specificity (95% CI)
Whole-blood Brix (%)	0.432 (0.201–0.676)	0.589	≤8.80	100.0	54.1–100.0	12.9	6.1–23.0
Fresh-serum Brix (%)	0.771 (0.608–0.911)	0.029	≤7.80	100.0	54.1–100.0	55.7	43.3–67.6
Frozen-serum Brix (%)	0.694 (0.470–0.882)	0.118	≤7.70	83.3	35.9–99.6	55.7	43.3–67.6
Total protein (g/dL)	0.986 (0.952–1.000)	<0.001	≤4.15	100.0	54.1–100.0	92.9	84.1–97.6
Globulin (g/dL)	0.981 (0.945–1.000)	<0.001	≤1.80	100.0	54.1–100.0	94.3	86.0–98.4
Weight change (%)	0.948 (0.876–0.993)	<0.001	≤8.27	100.0	54.1–100.0	82.9	72.0–90.8
CPV antibody assay value	0.907 (0.798–0.988)	0.001	≤761.0	100.0	54.1–100.0	72.9	60.9–82.8
GGT (U/L)	0.721 (0.407–0.957)	0.075	≤31.5	66.7	22.3–95.7	92.9	84.1–97.6

Lower values were defined as indicating FPT. Cutoffs maximize the Youden index in the present dataset and are exploratory. AUC values are apparent (within-sample) estimates. AUC confidence intervals were obtained from 10,000 stratified bootstrap samples. Confidence intervals for sensitivity and specificity are exact (Clopper–Pearson) intervals. AUC, area under the receiver operating characteristic curve; CI, confidence interval; FPT, failure of passive transfer; GGT, γ-glutamyl transferase.

**Table 5:** Bland–Altman agreement between paired refractometer measurements.

Comparison (Difference)	Mean Bias (%)	SD of Differences	95% CI for Bias	95% Limits of Agreement
Whole blood − fresh serum	−0.21	1.16	−0.47 to 0.06	−2.49 to 2.07
Whole blood − frozen serum	−0.12	1.20	−0.39 to 0.16	−2.46 to 2.23
Fresh serum − frozen serum	0.09	0.59	−0.04 to 0.22	−1.06 to 1.24

Bias was calculated as the first measurement minus the second measurement named in each comparison. Limits of agreement were calculated as mean bias ±1.96 SD. CI, confidence interval; SD, standard deviation.

## Data Availability

The original contributions presented in this study are included in the article. Further inquiries can be directed to the corresponding authors.

## Abbreviations

The following abbreviations are used in this manuscript:

4PL	Four-parameter logistic
ALB	Albumin
ALP	Alkaline phosphatase
AUC	Area under the receiver operating characteristic curve
CI	Confidence interval
CPV	Canine parvovirus
CPV-2	Canine parvovirus type 2
ELISA	Enzyme-linked immunosorbent assay
FPT	Failure of passive transfer
GGT	Gamma-glutamyl transferase
IgG	Immunoglobulin G
IQR	Interquartile range
OD	Optical density
PT	Passive transfer
ROC	Receiver operating characteristic
S/*P*	Sample-to-positive-control ratio
SD	Standard deviation
TP	Total protein
